# Computer-aided pattern scoring (C@PS): a novel cheminformatic workflow to predict ligands with rare modes-of-action

**DOI:** 10.1186/s13321-024-00901-5

**Published:** 2024-09-23

**Authors:** Sven Marcel Stefan, Katja Stefan, Vigneshwaran Namasivayam

**Affiliations:** 1 Department of Pathology, University of Oslo and Oslo University Hospital, Rikshospitalet, Sognsvannsveien 20, 0372 Oslo, Norway; 2 Lübeck Institute of Experimental Dermatology, Medical Systems Biology Division, Medicinal Chemistry and Systems Polypharmacology, University of Lübeck and University Medical Center Schleswig-Holstein, Ratzeburger Allee 160, 23538 Lübeck, Germany; 3 Department of Biopharmacy, Medical University of Lublin, Chodzki 4a, 20-093 Lublin, Poland; 4 Pharmaceutical Institute, Department of Pharmaceutical and Cellbiological Chemistry, University of Bonn, An der Immenburg 4, 53121 Bonn, Germany

## Abstract

The identification, establishment, and exploration of potential pharmacological drug targets are major steps of the drug development pipeline. Target validation requires diverse chemical tools that come with a spectrum of functionality, *e.g.*, inhibitors, activators, and other modulators. Particularly tools with rare modes-of-action allow for a proper kinetic and functional characterization of the targets-of-interest (*e.g.*, channels, enzymes, receptors, or transporters). Despite, functional innovation is a prime criterion for patentability and commercial exploitation, which may lead to therapeutic benefit. Unfortunately, data on new, and thus, undruggable or barely druggable targets are scarce and mostly available for mainstream modes-of-action only (*e.g.*, inhibition). Here we present a novel cheminformatic workflow—computer-aided pattern scoring (C@PS)—which was specifically designed to project its prediction capabilities into an uncharted domain of applicability.

**Supplementary Information:**

The online version contains supplementary material available at 10.1186/s13321-024-00901-5.

## Scientific contribution

The presented workflow tackles, for the first time, the challenge of data scarcity particularly focusing rare modes-of-action. In addition, the workflow and associated dataset provide new standards in the definition and application of criteria to rationalize drug candidate selection addressing important gaps in cheminformatics as well as computational and medicinal chemistry.

**Supplementary Information:**

The online version contains supplementary material available at 10.1186/s13321-024-00901-5.

## Introduction

Novel drug target identification and validation are two of the greatest challenges of medical life sciences today [1, 2]. Potential pharmacological targets identified from genomic, proteomic, and metabolomic analyses have a great chance to belong to the ~ 99% of disease-modifying proteins that cannot be targeted by small-molecules. Even ‘difficult-to-drug-targets’ [1]—which are technically not undruggable—come with a very limited number of mostly weakly potent modulators with very narrow and unsophisticated modes-of-action (*e.g.*, inhibition, antagonism, *etc.*). However, particularly the validation of novel drug targets requires collections of chemical tool compounds with a large spectrum of bioactivity [*e.g.*, partial inhibition/antagonism, inverse agonism, (non-)essential activation/agonism, (non-/un)competitive modulation, ortho- or allosteric binding, *etc.*). The therapeutic value of such agents may lie in extravagant and rare modes-of-action, as this is the case for modern therapeutic candidates [*e.g.*, covalent binders [3], degraders [4], polypharmacologicals [5], *etc.*). The development of such agents is a huge challenge in the light of target undruggability, but mandatory considering required originality and innovation of future therapeutics-to-be.

Chemical space harbors both originality and innovation of small-molecules, however, sophisticated fingerprints are needed to optimally handle the vastness of chemical space. These fingerprints need to be embedded in a computational workflow that enables the identification of the relevant chemical space, and at the same time, promotes their prediction into a far-reaching applicability domain. Recently, we have demonstrated that chemical patterns implemented in our novel drug discovery tool ‘computer-aided pattern analysis’ (‘C@PA’) are superior descriptors compared to those used in classical computational approaches such as pharmacophore modelling and similarity search [6]. Particularly, C@PA combined the sensitivity (prediction of true-positive hits) of pharmacophore modelling (62.5% *vs*. 60.4%) with the high specificity (identification of true-negative hits) of similarity search (90.8% *vs*. 87.3%), resulting in an overall biological hit rate of 21.7% [6]. Subsequent improvements of the model adjusting structural [7], bioactivity [8], and literary limits [9] resulted in an increase of the biological hit rates to 40.0–95.5% [7–9]. C@PA demonstrated an extensive scope of applicability by projecting its prediction capabilities far outside of the molecular-structural homogeneity the initial datasets were limited to [9–11]. While it showed strength toward uncharted ‘orphan target space’ [9], C@PA’s restriction lied in a narrow ‘mode-of-action space’ focusing inhibition only [6–11].

The current work describes a novel cheminformatic workflow to predict bioactive molecules with distinctive mode-of-action to tackle the lack of diversity in the ‘mode-of-action space’. The ATP-binding cassette (ABC) transporter ABCC1 was used as model system, as ABC transporter activation is a rare observation, however, reasonable knowledge about ABCC1 activation is available, and thus, a suitable workflow could be developed.

Therapeutically, ABCC1 activators would be ideal lead molecules in both cancer [12] and Alzheimer’s disease [13] research. Cancer cells have a much higher demand for reduced glutathione as per their *per se* higher level of cellular distress. The antioxidant reduced glutathione (GSH) is the prime substrate of ABCC1, which is often overexpressed in cancers due to the phenomenon of multidrug resistance [14]. Accelerating GSH efflux as anticancer strategy (‘collateral sensitivity’) has been suggested previously [15, 16]. Alzheimer’s brains, on the other hand, have an overload of amyloid-β (Aβ) proteins, and ABC transporters, including ABCC1, were discovered as major players in cerebral Aβ clearance, and thus, suggested as potential anti-Alzheimer’s drug targets [13].

## Results

### Generation and validation of input data

#### Compilation of data set

Compared to inhibition, ABC transporter activation can be considered as a rarely observed and reported mode-of-action. Recently, we summarized the entire knowledge on ABCC1 modulators [12, 17], from which we extracted compounds for the present study which demonstrated biological effects that allowed for the conclusion of an apparent ABCC1 activation. These effects included increased transport velocity/binding of either endo- or xenobiotic ABCC1 substrates, which resulted either in:

(i) a reduced intracellular concentration of the respective substrate in ABCC1-expressing cells; or
(ii) an increased intra-vesicular concentration of the substrate in ABCC1 inside-out membrane vesicles in presence of the apparent activator compared to control.

Compounds were excluded for further calculations if their effects were based on:

(i) promotion of ligand binding to ABCC1;
(ii) promotion of nucleotide binding to ABCC1;
(iii) promotion of nucleotide cleavage (increased ATPase activity);
(iv) induction of ABCC1 mRNA and/or DNA; and
(v) actin polymerization to promote ABCC1 trafficking to the plasma membrane.

In contrast to substrate binding, ligand binding is not directly associated with ligand transport, thus, these effects were precluded. At a first glance, the exclusion of ATPase activation sounds counterintuitive. However, activation of the ABCC1 ATPase itself (without other confirmatory data) is rather an indicator that the test compound is a substrate which is itself transported by ABCC1. As we anticipated transport activation of a substrate in presence of the test candidate, compounds that demonstrated increase of ABCC1 ATPase activity only had to be excluded.

**Fig. 1:**
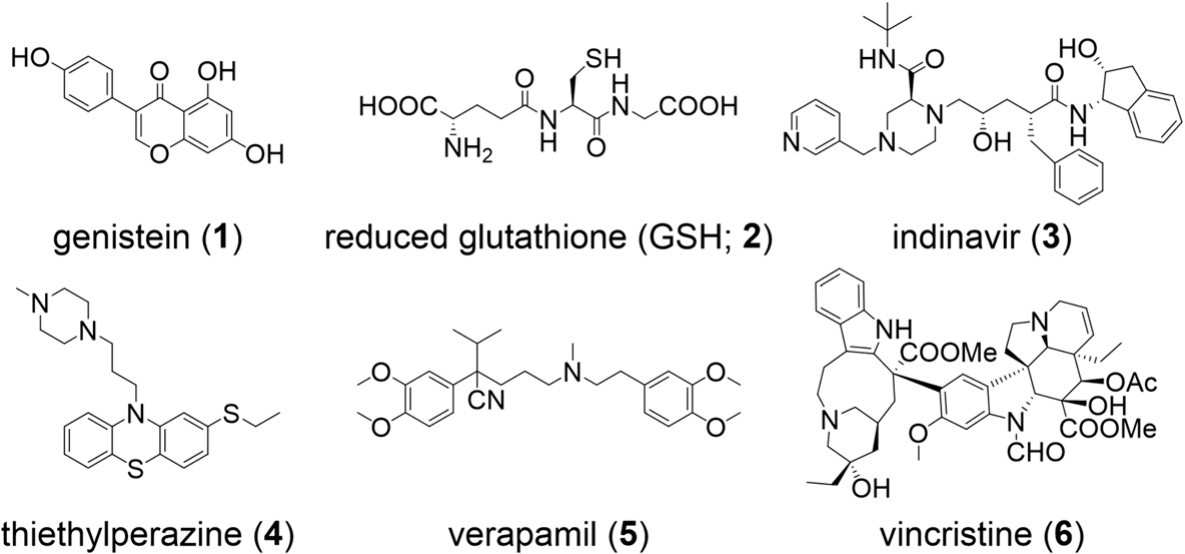
Examples of ABCC1 activators [18–23]

In total, 174 individual, qualified literature compounds from 26 reports between 1996–2017 were extracted for the present work, amongst which were, for example, genistein (**1**) [18, 19], GSH (**2**) [20], indinavir (**3**) [21], thiethylperazine (**4**) [22], verapamil (**5**) [18, 23], and vincristine (**6**) [18, 20] (all Fig. 1). Sheet 1, Additional file 1: Table A provides the entire list of 174 literature-documented ABCC1 activators.

#### Data records and FAIR-ification

The ABCC1 activation-focused dataset (Sheet 1, Additional file 1: Table A) consists of:

(i) individual identifiers for each compound, particularly:
  - an unique ID for each compound (‘ABCC1_Activation_Dataset_ID_0XXX’);
  - the original name of the compound as given in the original report(s);
  - a common abbreviation or synonym of the compound;
  - the systematic compound name according to the IUPAC nomenclature generated by ChemDraw Pro version 20.1.1.125;
  - the PubChem Compound ID retrieved from pubchem.ncbi.nlm.nih.gov (152 of 174 compounds);
  - the ChEMBL Compound ID retrieved from ebi.ac.uk/chembl (118 of 174 compounds);
  - the DrugBank Accession Number as retrieved from go.drugbank.com (30 of 174 compounds);
  - the IUPHAR/BPS Guide to Pharmacology Ligand ID as retrieved from guidetopharmacology.org (31 of 174 compounds);
  - the Chemical Abstracts Service (CAS) number as retrieved from commonchemistry.cas.org (72 of 174 compounds);
  - the chemical class of the compound;
  - the chemical class number of the compound;
(ii) molecular-structural and physicochemical descriptors, particularly:
  - the molecular structure of the compound conserved as SMILES code obtained either from
    1. the PubChem database (pubchem.ncbi.nlm.nih.gov); or
    2. manual drawing using ChemDraw Pro version 20.1.1.125 according to the 2D representation as given in the respective report(s) and/or supplementary information file(s);
  - the physicochemical properties as calculated with MOE version 2019.01, particularly:
    1. calculated octanol–water partition coefficient (CLogP);
    2. calculated solubility (CLogS);
    3. molecular weight (MW);
    4. molar refractivity (MR);
    5. topological polar surface area (TPSA);
  - molecular-structural properties as calculated by MOE version 2019.01, particularly:
    1. number of hydrogen-(H)-bond donors;
    2. number of H-bond acceptors;
    3. number of rotatable bonds;
    4. number of heavy atoms;
(iii) the bioactivity span in which activation of ABCC1 was observed; and
(iv) the assays and cell lines used to determine ABCC1 activation (assay 1, 2, …, 5), particularly:
  - efflux or uptake assays under referral to the used ABCC1 substrate;
  - cell line(s) used as ABCC1 host system(s);
  - cultivation protocol(s) and/or condition(s) used;
  - species of the cell line(s) used;
  - the Cellular Passport ID(s) of the cell line(s) as retrieved from cellmodelpassports.sanger.ac.uk;
  - the Cellosaurus ID(s) of the cell line(s) as retrieved from cellosaurus.org;
  - the American Type Culture Collection (ATCC) ID(s) of the cell line(s) as retrieved from atcc.org;
  - the digital object identifier(s), *i.e.*, PubMed ID(s) as retrieved from pubmed.ncbi.nlm.nih.gov.

#### Molecular-structural limitation of the dataset

**Fig. 2:**
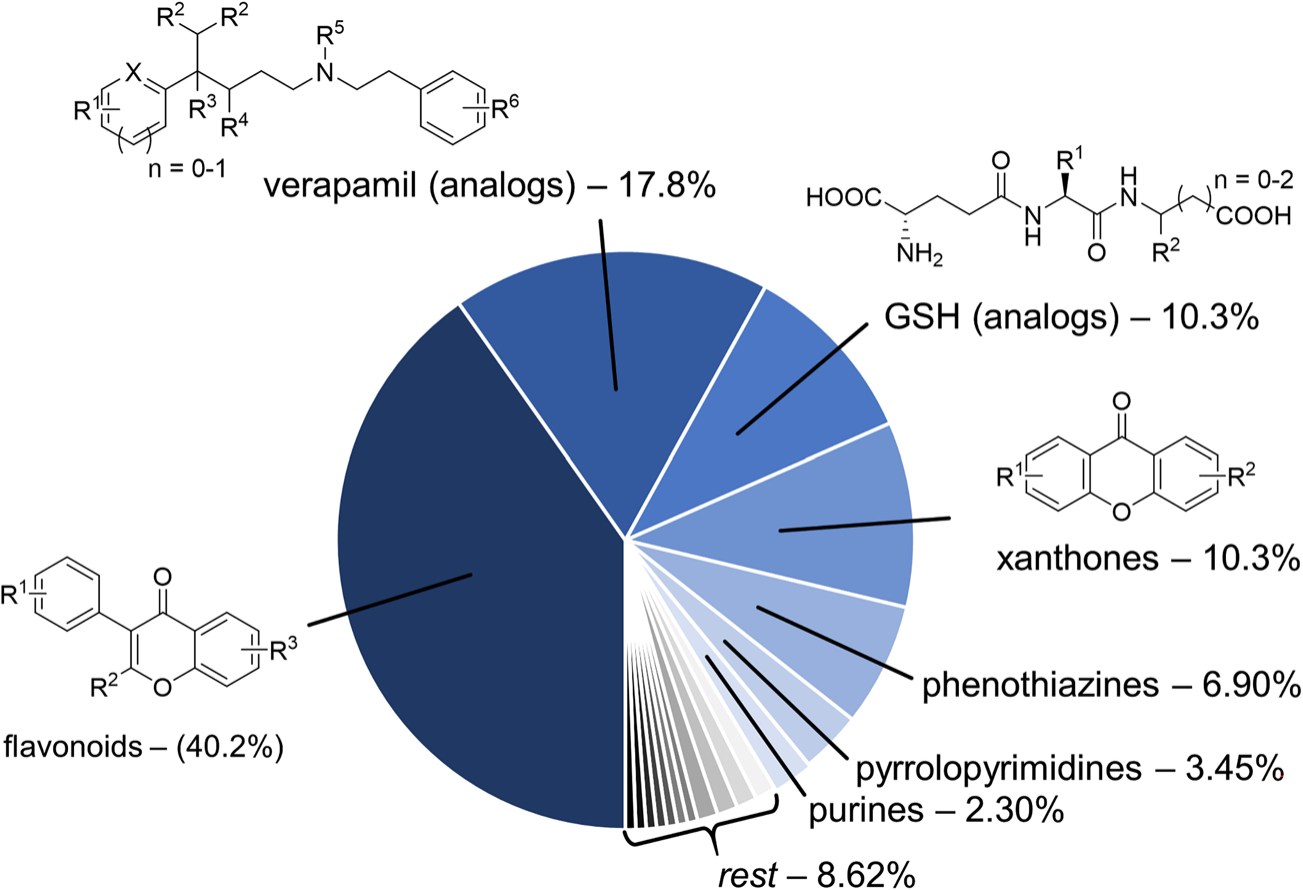
Visualization of molecular-structural homogeneity of the ABCC1 activation-focused dataset

The 174 ABCC1 activators consisted of 18 chemical classes, which suggests a high molecular-structural diversity of the dataset at first glance. However, the 174 compounds were unequally distributed amongst these 18 classes as demonstrated in Fig. 2. The largest portion of the entire dataset constituted flavonoids (40.2%), verapamil analogs (17.8%), GSH analogs (10.3%), xanthones (10.3%), phenothiazines (6.90%), pyrrolopyrimidines (3.45%), and purines (2.30%)—while the other 15 compounds (8.62%) consisted of 11 different chemical classes. This molecular-structural homogeneity resulted from few medicinal chemistry efforts to generate small (but significant) compound libraries of individual chemical classes (*e.g.*, flavonoids [24], verapamil analogs [25], or GSH analogs [26]). In total, 134 of the 174 compounds (77.0%) are derivatives / analogs. These molecular-structural redundancies contributed majorly to the molecular-structural homogeneity of the ABCC1 activation-focused dataset.

#### Bioactivity limits of the dataset

**Fig. 3:**
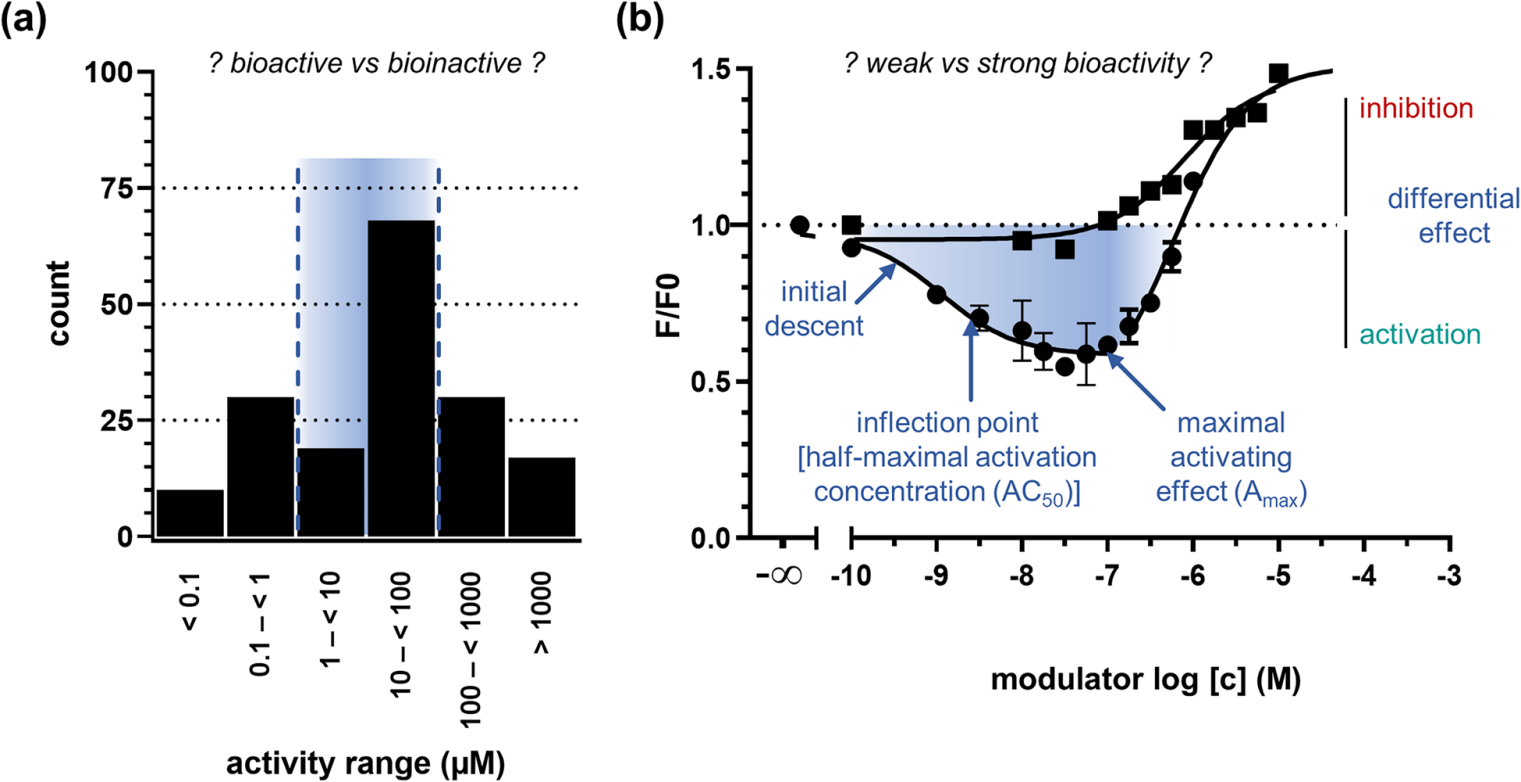
Distribution (**a**) of bioactivity values of literature-documented ABCC1 activators; the lowest value of the bioactivity span of the compounds was considered. A representative concentration-effect curve of an activator (closed circles) and a reference inhibitor (closed squares) is shown (**b**) outlining important curve parameters. Curve taken and adapted from reference [27]

Apart from the molecular-structural homogeneity of the ABCC1 activation-focused dataset, the diversity of reported bioactivities was another challenge. As can be seen in Fig. 3a, the bioactivities reported ranged from nanomolar (nM) to millimolar (mM) concentrations (0.01–5000 µM) [18, 27], which exacerbates the identification of a threshold to differentiate between bioactive and bioinactive. A threshold at higher concentrations increases the molecular-structural diversity of the input compounds for the computational prediction, however, lowers significantly the potency of the output compounds.

Moreover, most activity values were reported as single point measurements only instead of half-maximal concentrations as necessary to develop computational models. These single point concentrations are difficult to interpret as it is unknown which concentration-effect [initial descent, inflection point (half-maximal activation concentration, AC_50_), maximal effect (A_max_ [28]), *etc*.; Fig. 3b] of the concentration-effect curve has been observed. This is even more true for differential effects, *i.e.*, opposing effects at different concentrations, which have particularly been shown for ABCC1 activators (*e.g.*, activation at lower concentrations and inhibition at higher concentrations [27]; Figs. 3b).

#### Physicochemical and molecular-structural validation 

**Fig. 4:**
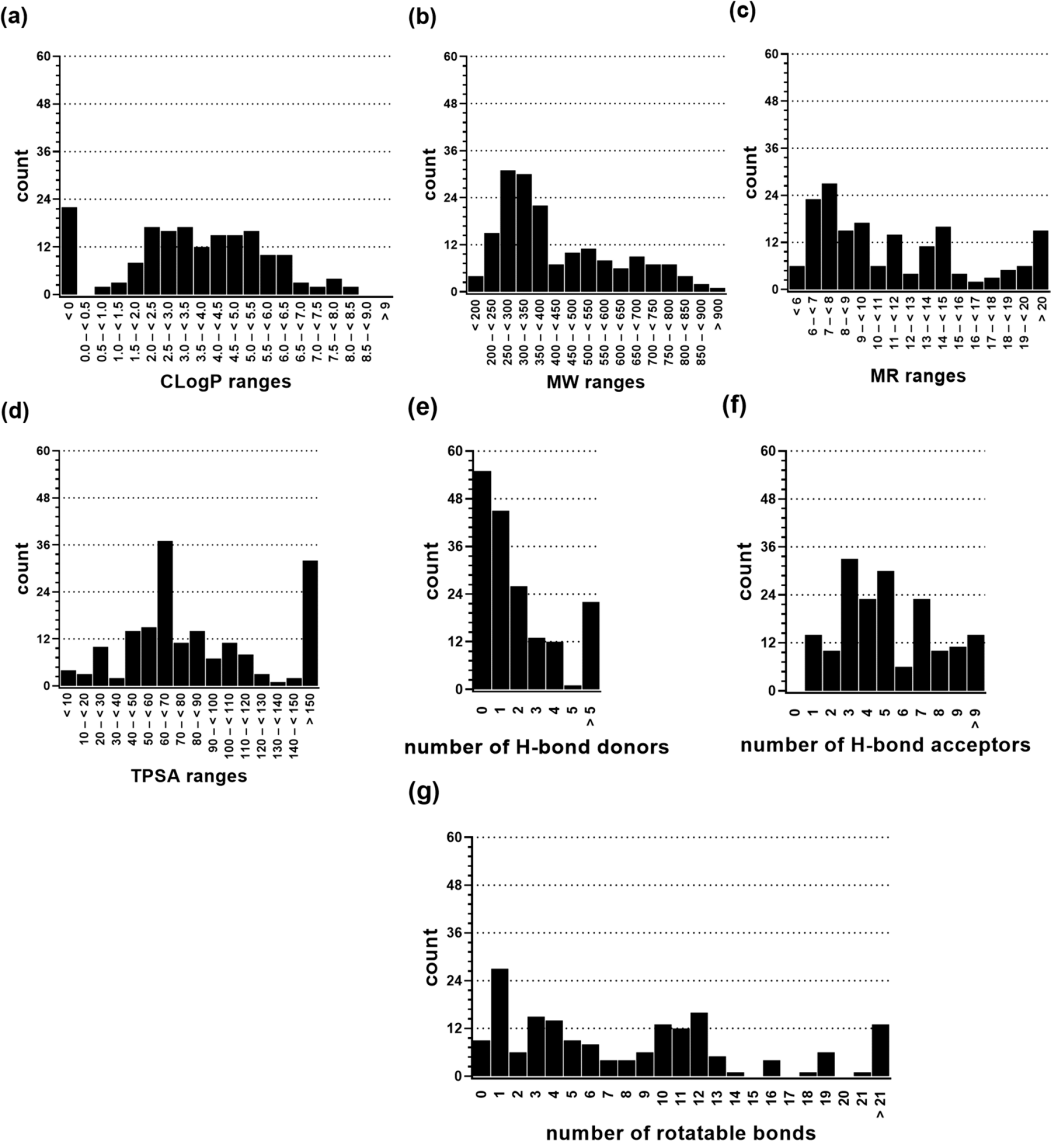
Distribution of physicochemical and molecular-structural attributes of the 174 molecules of the ABCC1 activation-focused dataset as determined by MOE version 2019.01. **a** Calculated octanol–water partition coefficient (CLogP). **b** Molecular weight (MW). **c** Molar refractivity (MR). **d** Topological polar surface area (TPSA). **e** H-bond donors. **f** H-bond acceptors. **g** Rotatable bonds

The general validity of datasets is expressed by balanced distributions of physicochemical [*e.g.*, calculated octanol–water partition coefficient (CLogP), molecular weight (MW), molar refractivity (MR), and topological polar surface area (TPSA)] and molecular-structural [*e.g.*, hydrogen (H-)bond donors, H-bond acceptors, and rotatable bonds] parameters. As can be seen in Fig. 4, gaussian distributions of the assessed physicochemical and molecular-structural parameters for the 174 compounds could be observed. This equal distribution is underpinned by median and mean values that are well-aligned as shown in Table 1. However, particularly molecules at the lower and upper edges of the scales were pronounced, which can be explained by the rather low number of 174 compounds within the dataset, which has been observed in other datasets before [10, 11, 29].

**Table 1:** Median and mean values of the physicochemical properties CLogP, MW, MR, and TPSA, as well as the molecular-structural attributes H-bond donors, H-bond acceptors, and rotatable bonds of the 174 compounds of the ABCC1-focused dataset as determined by MOE version 2019.01

	CLogP	MW	MR	TPSA	H-bond donor	H-bond acceptors	Rotatable bonds
Median	3.51	366.93	9.80	72.46	1	5	6
Mean	3.15	429.53	11.51	90.40	2.02	5.32	8.24

### Summary of dataset-related challenges

The validation of the ABCC1 activation-focused dataset allowed for the conclusion of the following impediments:

(i) the dataset is very small (174 compounds) and based on a very limited number of reports only (26);
(ii) identified activators were almost always serendipitous findings with no further elucidation of the structure–activity relationships or the general background of the mode-of-activation of these compounds;
(iii) the molecular-structural homogeneity reduces the relevant chemical space and the discriminatory potential of the output fingerprint;
(iv) the molecular-structural homogeneity impacts the molecular-structural diversity, and thus, the originality and innovation of predicted output molecules; and
(v) the diversity in the bioactivity landscape impedes the targeted development of potent activators of sufficient molecular-structural diversity.

The stated impediments are valid for any experimental and explorative target (or target combination), and the following methodological steps demonstrate how to gain novel and innovative compounds from very limited structural, molecular, and functional knowledge.

### Generation of output data

#### Basic scaffold search 

In a first step, the set of 174 ABCC1 activators was analyzed for common core features (‘basic scaffolds’) that frequently occurred. By applying SARreport [6, 30] implemented in MOE version 2019.01, three basic scaffolds could be identified:

(i) chromone;
(ii) xanthone; and
(iii) phenothiazine

However, SAReport was not able to classify 75 of the 174 compounds. Amongst these 75 non-classified compounds were 31 verapamil and 18 GSH analogs, which are structurally indistinctive, and thus, hard to classify. The other 26 molecules consisted mostly of heteroaromatic scaffolds which prompted us to re-analyze this set of 75 non-classified compounds with SAReport. By this measure, two more basic scaffolds could be identified:

(iv) purine; and
(v) 9-deazapurine.

**Fig. 5:**
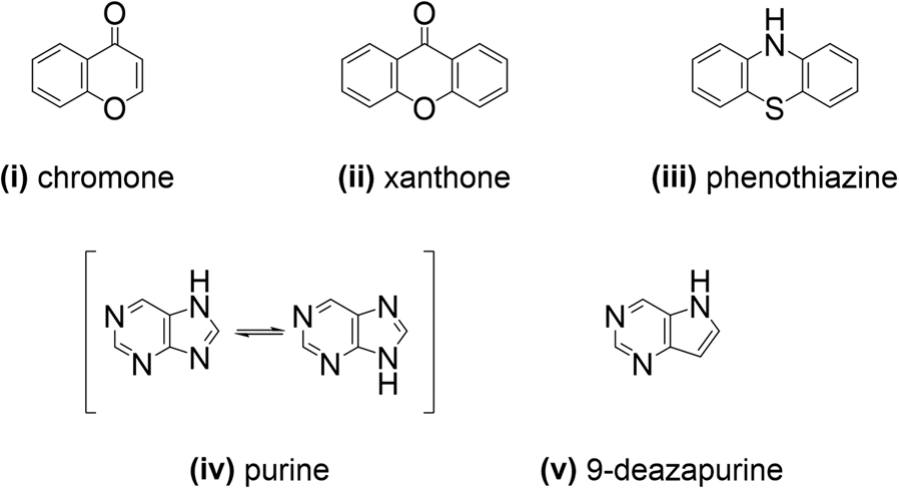
Identified basic scaffolds shared by a large fraction (99 compounds) of the 174 ABCC1 activators using SARreport [6, 30] implemented in MOE version 2019.01

Figure 5 provides the molecular formulae of the identified basic scaffolds.

#### Chemical pattern analysis

Chemical patterns are superior fingerprints if embedded in a sophisticated workflow [6–11]. The initial pattern analysis framework of C@PA worked with a catalog of 308 molecular substructures, of which only 162 were active (= present) amongst the set of molecules that was analyzed [6]. As a second step of the herein described workflow, we analyzed each of the 174 ABCC1 activators for molecular substructures considering both data records of already reported substructure catalogs [6, 10, 11, 29] as well as visualization and generation of novel substructures. We identified in total 243 substructures that accurately described the entire set of 174 molecules, of which 16 have not been described earlier [6, 10, 11, 29]. Sheet 2, Additional file 1: Table B lists all 243 substructures used to generate the activation fingerprints.

#### Activation fingerprints

**Fig. 6:**
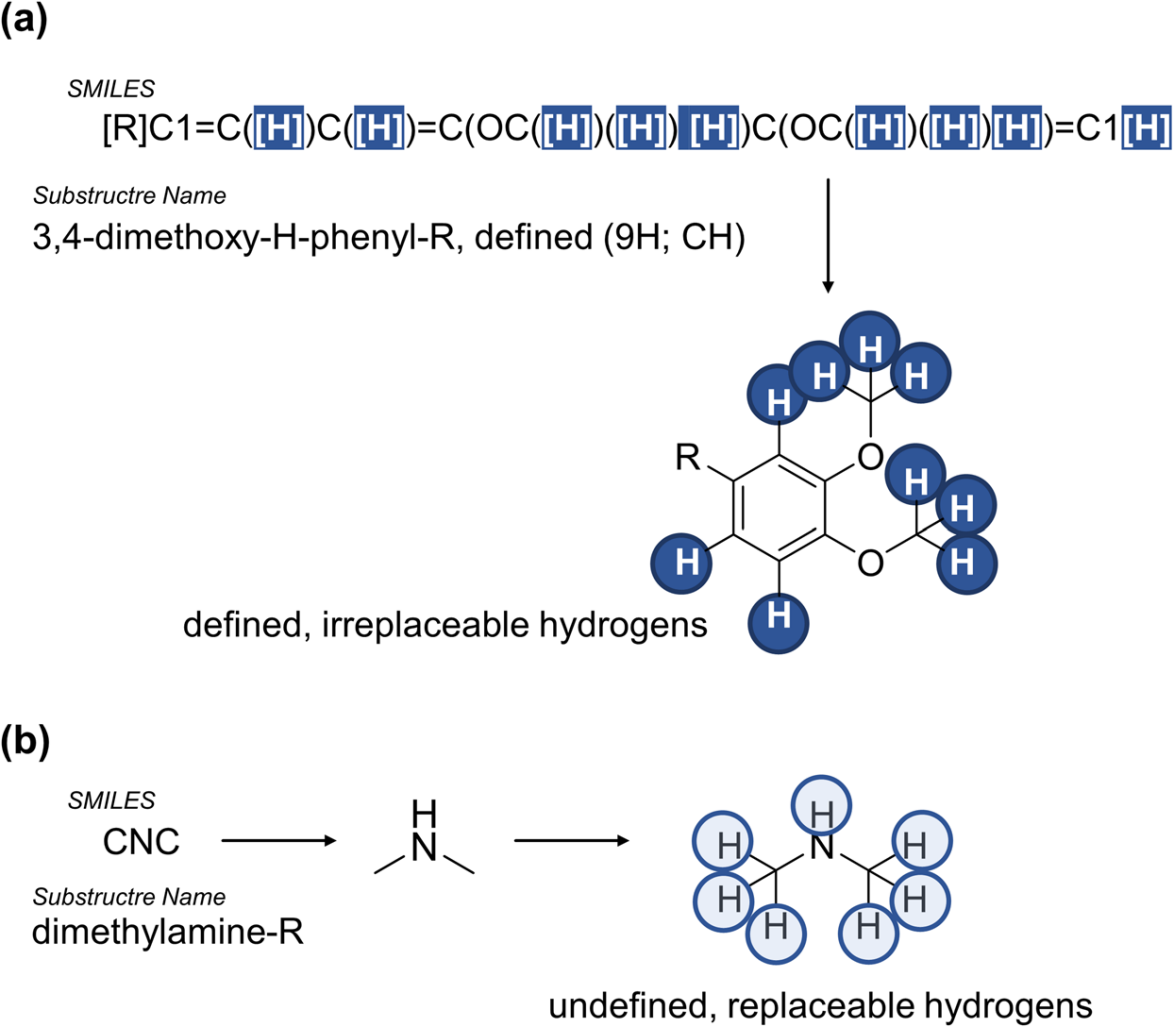
Comparison between the highly specific but less competitive substructure 3,4-dimethoxyphenyl (**a**) and the highly unspecific but strongly competitive substructure dimethyl amine (**b**)

To translate the 243 molecular substructures into biological meaning, a scoring scheme was applied to generate different chemical pattern-based fingerprints which were subsequently used for virtual screening purposes. Four fingerprints were generated to acknowledge both the homogeneity of the ABCC1 activation-focused dataset and the heterogeneity of the 243 substructures:

(i) fingerprint I: the percentage of the 174 compounds was calculated in which each of the 243 substructures occurred in, and the 243 substructures were ranked from most abundant (100–90%) to least abundant (< 10–0%). For further calculations, substructures were used only if they were present in at least 20% of the 174 compounds (exceptions applied, see below);
(ii) fingerprint II: the set of 174 compounds consists of many structurally very similar molecules (*e.g.*, 70 flavonoids, 31 verapamil derivatives, and 18 xanthones). Thus, certain molecular substructures may be overrated if their analysis was based solely on their percentage occurrence amongst the 174 compounds. To enable a proper consideration of alternative substructures and to allow for a different ranking order, the 174 compounds were first allocated into 18 different chemical classes, and the percentage occurrence of each of the 243 substructures within these 18 chemical classes was analyzed. Here again, the 243 substructures became ranked from most abundant (100–90% of classes) to least abundant (< 10–0% of classes), and substructures were used only if they were present in at least 20% of the 174 compounds (exceptions applied, see below);
(iii) fingerprint III: one of the major conclusions of C@PA was that the definition of (irreplaceable) hydrogens (defined as ‘[H]’ in SMILES codes) and their positioning in substitution patterns with other substituents provide a critical discriminatory potential for the virtual screening process between potentially bioactive and bioinactive molecules [6–10, 29]. Systematic inclusion of defined [H] drastically increases the structural diversity of the substructure catalog, *e.g.*, 3,4-dimethoxyphenyl (Fig. 6a). However, this makes the respective substructure less competitive compared to very general substructures, *e.g.*, dimethyl amine (Fig. 6b) in terms of occurrence. General substructures with undefined hydrogens which may be replaced by any other atom according to SMILES rules are more likely to be found within the analyzed set of compounds, and thus, these substructures may be overrated due to their molecular-structural unspecificity. To increase the value of molecular-structurally specific substructures, we calculated the ‘average flexibility’ of all 243 substructures by dividing the number of non-defined hydrogens by the number of heavy atoms within the respective substructure. The quotient was used as denominator to divide the fraction value as a result of fingerprint I for each substructure, and the resultant mathematical values associated with the respective substructures were ranked from largest (1.77–0.90) to smallest (< 0.01); and
(iv) fingerprint IV: the average flexibility quotient was also used as denominator to divide the fraction value as a result of fingerprint II for each substructure, and the resultant mathematical values associated with the respective substructures were ranked from largest (1.33–0.90) to smallest (< 0.1).

**Fig. 7:**
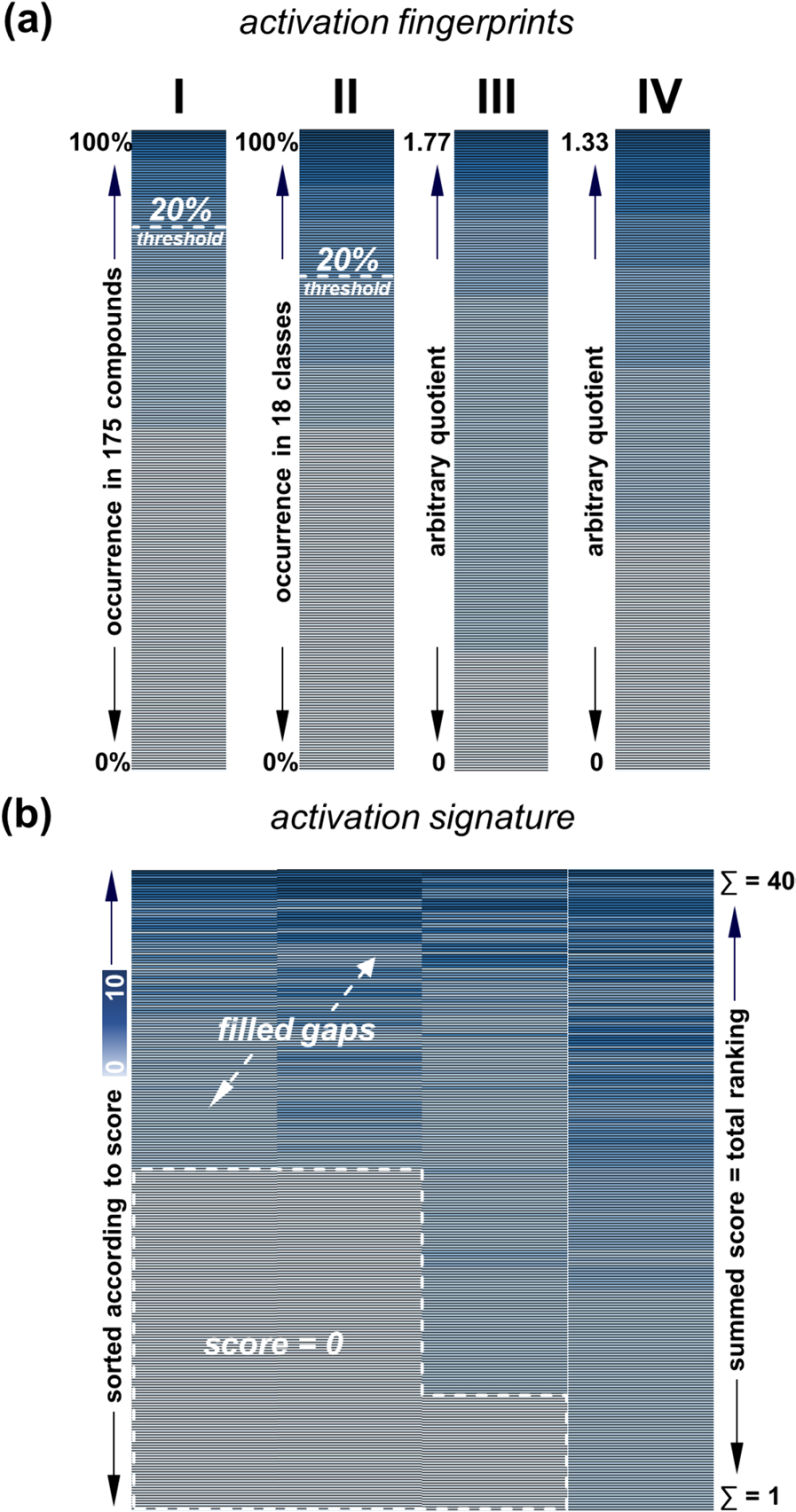
Visualization of the four distinct fingerprints (**a**) and the concluded signature (**b**) used for virtual screening of chemical space

Sheet 2, Additional file 1: Table B provides the above-mentioned information, particularly:

(i) individual substructure identifiers (‘Substructure_0XXX’) under consideration of previously established substructure labels [10, 11, 29] according to FAIR principles;
(ii) the trivial name of the substructures;
(iii) the molecular structure of the substructures conserved as SMILES code;
(iv) number of hits within the 174 compounds for each substructure;
(v) percentage occurrence of respective substructure in 174 compounds (basis for fingerprint I);
(vi) number of hits within the 18 chemical classes for each substructure;
(vii) percentage occurrence of respective substructure in 18 chemical classes (basis for fingerprint II);
(viii) total number of hydrogens per substructure;
(ix) number of defined hydrogens (‘[H]’ in SMILES codes) per substructure;
(x) number of variable hydrogens per substructure [*i.e.*, value of (viii) minus value of (ix)];
(xi) number of heavy atoms per substructure;
(xii) calculated average flexibility per heavy atom per substructure [*i.e.*, value of (x) divided by value of (vi)];
(xiii) quotient of occurrence within 174 compounds and average flexibility [*i.e.*, value of (v) divided by value of (xii); basis for fingerprint III]; and
(xiv) quotient of occurrence within 18 chemical classes and average flexibility [*i.e.*, value of (vii) divided by value of (xii); basis for fingerprint IV];

Figure 7a visualizes the generated fingerprints used in this work.

### Activation signature

The four fingerprints contain the same 243 substructures in varying orders depending on the focus of the respective fingerprint. To allow for the equal use of all four fingerprints, scores between 10 and 0 were allocated to each of the ranked 243 substructures in each fingerprint (1.77–0.90 = 10; < 0.90–0.80 = 9; < 0.80–0.70 = 8; < 0.70–0.60 = 7; < 0.60–0.50 = 6; < 0.50–0.40 = 5; < 0.40–0.30 = 4; < 0.30–0.20 = 3; < 0.2–0.10 = 2; < 0.10–0.01 = 1; < 0.01 = 0).

As indicated above, substructures of fingerprints I–II were considered only if they were at least 20% present (score: ‘3’), all other substructures were considered with a score of ‘0’. However, certain substructures in fingerprints III–IV were represented with scores above ‘2’ despite their allocated score of ‘0’ in fingerprints I–II. In these cases, these value gaps were filled with the respective scores (either ‘1’ or ‘2’) of fingerprints I–II. This measure promoted the consideration of alternative substructures associated with ABCC1 activation using the average flexibility. Thereby, the global diversity of the applied substructures increased, tackling the challenges provided by the input data.

Figure 7b shows the activation signature after ranking of the privileged substructures according to the individual fingerprint values (total scores 40–1), while Sheet 3, Additional file 1: Table C provides the entire signature with 243 substructures. From the activation signature, 93 privileged substructures [31] could be identified that were designated as ‘primary positive substructures’ that fulfilled the following criteria:

(i) occurrence in fingerprint(s) I and/or II by ≥ 20%; or
(ii) occurrence in fingerprint(s) III and/or IV with a fraction value of at least 0.2 and occurrence in fingerprint(s) I and/or II by ≥ 10%.

All other 150 substructures were considered as ‘secondary positive substructures’. Sheet 3, Additional file 1: Table C shows both primary and secondary positive substructures.

#### Virtual screening and rationalized manual selection

The Enamine Real Space virtual compound library (enamine.net) was used for virtual screening consisting of 15,547,092 compounds. This virtual compound library was downsized by searching for the five basic scaffolds (chromones: 5713; xanthones: 0; phenothiazines: 684; purines: 39,356; 9-deazapurines: 1697), resulting in 47,445 relevant compounds (5 molecules had two basic scaffolds) summarized in Sheet 4, Additional file 1: Table D.

**Fig. 8:**
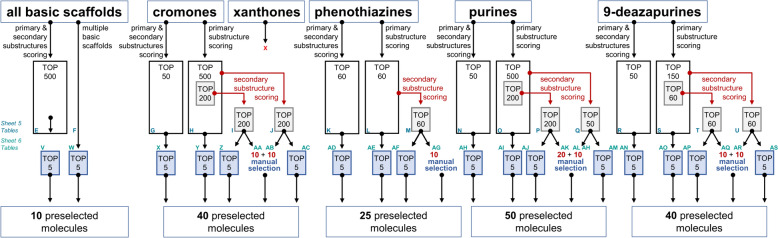
Visualization of the rationalized manual selection scheme to filter for potentially bioactive ABCC1 activators. In total, 47,445 molecules were subject to a scoring according to primary and/or secondary positive substructures, and several different lists of compounds containing chromones, phenothiazines, purines, and 9-deazapurines were generated from which the top score molecules were selected as outlined above

These 47,445 relevant compounds were subjected to a selection scheme that we designated as ‘rationalized manual selection’. This first-in-field scheme shall allow for the selection of the potentially best molecules with the greatest systematic, and at the same time, allow for the acknowledgement of empirical experience of researchers by small-scale manual selection. Hit candidates were selected under consideration of different scoring lists, taking primary and/or secondary positive substructures into account, which is visualized in Fig. 8.

Sheet 5, Additional file 1: Tables E–U provide the differently ranked lists of qualified compounds from which a selection of potential hit candidates took place. In total, 165 potential hit candidates were chosen which are summarized in Sheet 6, Additional file 1: Tables V–AS. These molecules were visualized and shortlisted according to their lipophilicity (CLogP) and molecule weight (MW) with the aim of a balanced selection. Finally, 75 were purchased from Enamine of which 49 (compounds **7**–**55**) were delivered (Sheet 7, Additional file 1: Table AT) for biological evaluation, while 26 were not available (Sheet 7, Additional file 1: Table AU).

### Biological model verification

#### Screening for ABCC1 activation

**Fig. 9:**
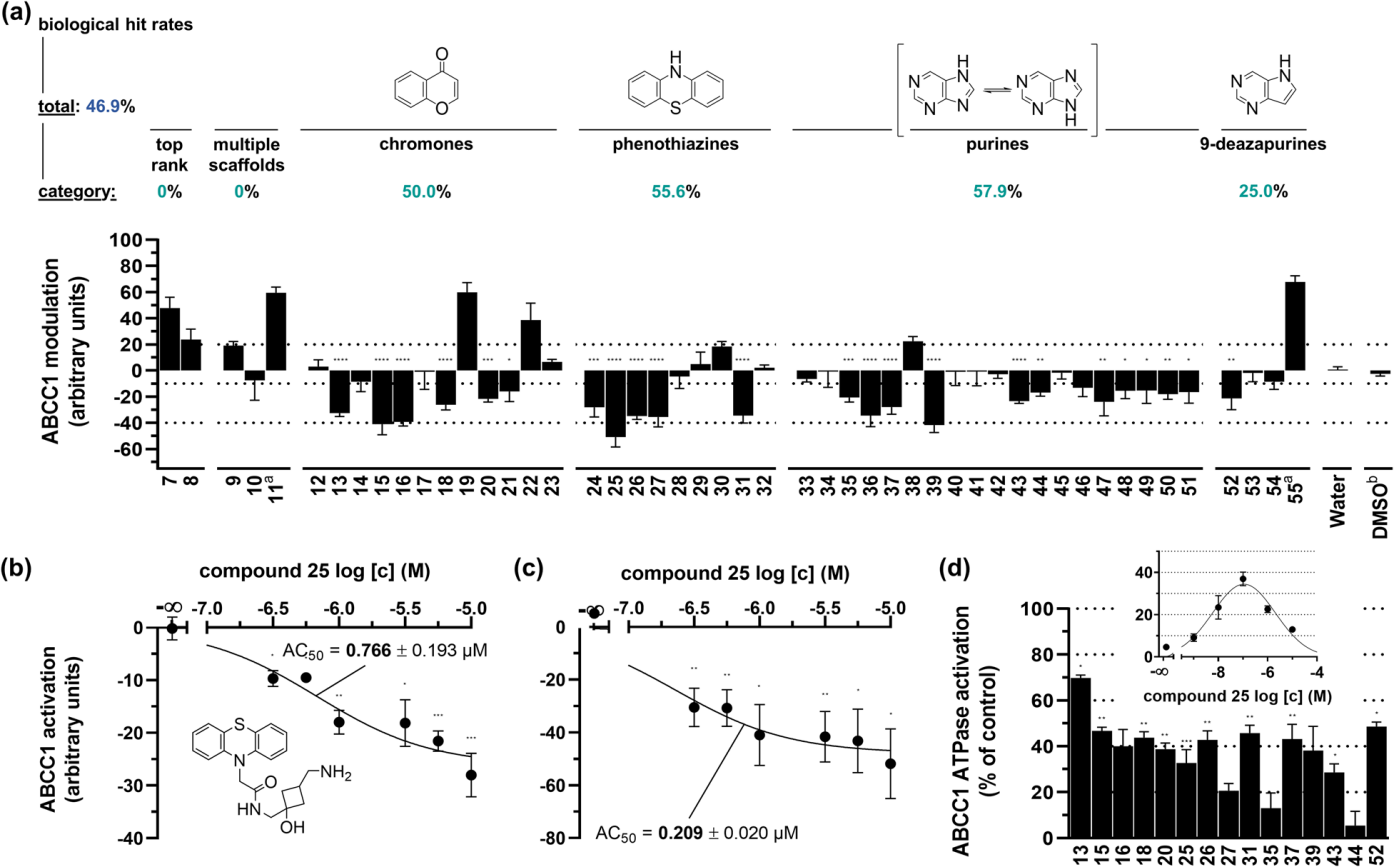
Biological assessment of compounds **7**–**55** for ABCC1 activation. **a** Initial screening results applying a fluorescence-based daunorubicin assay using ABCC1-expressing H69AR cells as described before [27]. Normalization of data was performed using the biological effects of the ABCC1 reference inhibitor 4-(4-(benzo[d][1,3]dioxol-5-ylmethyl)piperazin-1-yl)-6,7,8,9-tetrahydropyrimido [4,5-b]indolizine-10-carbonitrile at 10 µM (100%) [32] and pure cell culture medium (0%), respectively. The biological hit rate was calculated taking the significant effects into account only. **b**, **c** In-depth analysis of lead compound **25** at varying concentrations in the daunorubicin **b** [27] and rhodamine 123 **c** [33] assays. **d** Determination of the vanadate-sensitive ATPase assay as described before in absence and presence of selected test compounds (0.1 µM) [33]. Shown are mean values ± standard error of the mean (SEM) of at least three independent experiments, and significance was calculated using a t test considering a *p* value of 0.05 as significant; *p* ≤ 0.05; *p ≤ 0.01: **p ≤ 0.001: ***p < 0.001: ****; ^a^Inhibitory activity of these compounds already reported in reference [13]; ^b^water with DMSO content similar to test compounds (0.1%)

The largest part of the input molecules demonstrated apparent ABCC1 activation by promoted extrusion of ABCC1 substrates from ABCC1-expressing cell [12]. Thus, the output molecules were expected to reproduce these biological effects. We used a daunorubicin accumulation assay [27] and ABCC1-expressing H69AR cells to assess compounds **7**–**55** compared to a reference inhibitor of ABCC1 [32], and the results are shown in Fig. 9a.

Strikingly, 57.1% of the assessed compounds showed a tendency to lower intracellular daunorubicin concentrations within the cells, which indicates ABCC1 activation, and the effect of 46.9% of the compounds was significant (p ≤ 0.05). This is a surprisingly high hit rate considering that ABCC1 activation has never been systematically analyzed or anticipated, and that ABC transporter activation is a rather rare mode-of-action [12, 13].

To a surprise, none of the analyzed top rank compounds showed the intended effect, however, this can be explained by the low number of compounds analyzed. In addition, none of the compounds bearing two basic scaffolds showed an activatory effect. The reason could be that none of the input compounds had a similar composition, and that two (sterically demanding) basic scaffolds potentially counteract any activating effect coming from each scaffold. On the other hand, the hit rates for chromones (50.0%), phenothiazines (55.6%), and purines (57.9%) were rather comparable, while the hit rate of 9-deazapurines (25.0%) was lower. The reason here could also be the low sample size due to the undeliverability of 9-deazapurines in contrast to the other basic scaffold-containing molecules.

#### In-depth analysis of selected compounds

Compound **25**—a phenothiazine—showed the strongest apparent ABCC1 activation. This effect was concentration dependent, and in addition, could be reproduced in an alternative assay using rhodamine 123 as a substrate of ABCC1 [33] . The half-maximal activation concentration (AC_50_) [28] values of compound **25** were 0.766 µM (daunorubicin; Fig. 9b) and 0.209 µM (rhodamine 123; Fig. 9c).

ABC transporters consume energy (*i.e.*, ATP) in order to facilitate the active efflux of substrates. Although ATPase data have been excluded from the input data as sole ATPase activation is rather an indicator that the test compound is a substrate which is itself transported by ABCC1. However, together with confirmatory data such as in Fig. 9a–c, ATPase data may even substantiate the hypothesis. Here, ABCC1 activation should reflect in an increased activity of the ABCC1 ATPase. This was the case for most selected candidates with pronounced effects in the screening (Fig. 9d). The ABCC1 ATPase-activating effect of lead compound **25** was concentration dependent. These data provide a prospect of the discovery of more potent activators of ABCC1 based on compound **25**, and Sheet 8, Additional file 1: Table AV lists 58 potentially active molecules for future synthetic endeavors.

#### Screening for ABCB1 and ABCG2 activity

All compounds were also screened against the multidrug transporters ABCB1 and ABCG2. While 23 compounds showed slight apparent activation of ABCB1 in a daunorubicin assay, only one showed a similar effect toward ABCG2 in a pheophorbide A assay (Additional file 2: Fig. S1). These results, however, spark interest in the light of potential multitarget activation which warrants further investigation.

## Conclusions

Small-molecules play an important role in the establishment of novel potential pharmacological drug targets of the future. They may not only serve as lead structures for future therapeutics, but also as chemical tools to assess the functionality of these targets—explaining target physiology and biological effects on a molecular level. Here, a spectrum of mechanisms-of-action is needed for a proper kinetic and functional assessment of these targets. Unfortunately, new targets go along with data scarcity. The herein described workflow dissected the steps necessary to take to tackle this challenge and to address this gap in modern cheminformatics:

(i) the detailed analysis of the input data and the sophisticated, rational, and balanced selections allowed for a high biological hit rate at a comparably low number of analyzed compounds (= low-throughput suitable). This aspect makes C@PS an interesting application even for smaller laboratories with financial constraints saving precious resources such as time, funds, and personnel;
(ii) specifically the signature consisting of four different fingerprints promoted C@PS’s robustness, allowing for parallel approaches of different foci with still high hit rates;
(iii) the provided hit molecules reproduced the exact same biological effects of the structurally and functionally very limited input molecules;
(iv) both the molecular-structural and bioactivity limits could be overcome, providing novel molecules with moderate to strong effect within their exerted (and anticipated) mode-of-action. Particularly the exploitation of chemical space—including its novelty and originality—is a strength of C@PS;
(v) specifically the elucidated substructures address the question of the molecular background of ABCC1 activation in particular, and potentially ABC transporter activation in general. As general principles of the structural and functional nature between protein families and species exist [34, 35], these conclusions may apply to this (and other) modes-of-action as well.

C@PS and its associated dataset (Additional file 1) provide inclusive molecular-structural and functional knowledge with an applicability domain beyond its limitations related to compound-, bioactivity-, and target-related constraints. With now 733 substructures available [8], pattern analysis is able to accurately describe molecule populations of various sizes, and more, to extract relevant fingerprints for prediction and projection into unknown spaces [6–9, 11]. Thus, it is an ideal application to explore other, undruggable targets for chemical tools with rare and innovative—and potentially therapeutically preferable—modes-of-action.

## Methods

### Data mining and curation to compile ABCC1 activation-focused dataset

All molecules retrieved from our previous report [12] were filed using Microsoft Excel 2016 and associated content such as identifiers and annotations acquired from public databases, *i.e.*, PubChem (pubchem.ncbi.nlm.nih.gov), ChEMBL (ebi.ac.uk/chembl), DrugBank (go.drugbank.com), IUPHAR/BPS Guide to Pharmacology (guidetopharmacology.org), Chemical Abstracts Service (commonchemistry.cas.org), Cell Model Passports (cellmodelpassports.sanger.ac.uk), Cellosaurus (cellosaurus.org), American Type Culture Collection (atcc.org), and PubMed (pubmed.ncbi.nlm.nih.gov). The physicochemical properties calculated octanol–water partition coefficient (CLogP), molecular weight (MW), molar refractivity (MR), and topological polar surface area (TPSA), as well as the molecular-structural properties hydrogen-(H)-bond donors, H-bond acceptors, and rotatable bonds were generated using Molecular Operating Environment (MOE) version 2019.01. Molecular structures of molecules and substructures were visualized using ChemDraw Pro version 20.1.1.125, MOE version 2019.01, and InstantJChem version 21.13.0, and if returned without error, considered as valid.

### Pattern analysis: Basic scaffold search as well as substructure generation and search

The Structure–Activity-Report (SAReport) tool [30] implemented in MOE version 2019.01 was used to search the 174 ABCC1 activators for their basic scaffolds as reported earlier [6]. A chemical pattern catalog was used based on our previous reports [10, 11, 29] complemented by manual visualization and generation of customized substructures using ChemDraw Pro version 20.1.1.125 and a heavy atom distribution scheme as earlier [7]. The chemical patterns were searched for in the ABCC1 activation-focused dataset using the query search function of InstantJChem version 21.13.0, and the relative distribution was calculated using Microsoft Excel 2016.

### Biological assessment

#### Cell culture

The ABCC1-expressing cell line H69AR was purchased from American Type Culture Collection (ATCC; No. CRL-11351) and cultivated in RPMI-1640 media complemented with 20% fetal bovine sera (FBS), streptomycin (50 µg/µL), penicillin G (50 U/mL), and L-glutamine (2 mM) [6–8, 13, 32]. The cells were stored under liquid nitrogen (media/DMSO: 90%/10%), and cultivated at 37 °C and 5% CO_2_-humidified atmosphere. Passaging was performed using a trypsin–EDTA solution (0.05%/0.02%) at confluence of ~ 90%, followed by washing, centrifugation (266 × *g*, 4 °C, 4 min), re-suspending in fresh media, and seeding into new cell culture flasks and/or 96-well plates. The cells were counted using a Scepter handheld automated cell counter (60 µM capillary sensor; EMD Millipore). Details on ABCB1-and ABCG2-expressing cells and cell culture parameters have been reported previously [36].

#### Functional assessment of ABCC1 activity

Daunorubicin [6, 8, 13, 32, 33] and rhodamine 123 [33] assays were performed as reported earlier. Clear 96-well plates were loaded with 20 µL test compounds at 10 µM (screening) or various concentrations (in-depth analysis) followed by addition of 160 µL cell suspension (45,000 cells/well in colorless RPMI-1640 without further supplements. The cells were pre-incubated with the compounds for 30 min before adding 20 µL of the fluorescence dye (daunorubicin: 30 µM; rhodamine 123: 3 µM). The average steady-state fluorescence per well after incubation [daunorubicin: 180 min; rhodamine 123: 120 min; excitation: 488 nm; emission: 695/50 (daunorubicin) and 530 nm (rhodamine 123)] was determined applying  an Attune NxT (Invitrogen). Details on ABCB1- and ABCG2-based functional assays have been reported previously [36].

#### Functional assessment of ABCC1 ATPase activity

A vanadate-sensitive ATPase assay was performed as already described before [33]. [3-(*N*-morpholino)propanesulfonic acid-(MOPS)-Tris (40 mM; pH 7.0), KCl (50 mM), dithiothreitol (2 mM), EGTA-Tris (500 µM; pH 7.0), sodium azide (5 mM), ouabain (1 mM) constituted the reaction mixture 10 µg of the ABCC1 membrane preparation (2 mg/mL) was exposed to. The test compounds (20 µL; screening: 0.1 µM; compound **25**: various concentrations with a final DMSO concentration below 1%), GSH (5 mM), or orthovanadate (control; 1 mM) were added. MgATP (3.3 mM in water) started the reaction for 60 min at 37 °C. SDS (5%) stopped the reaction followed by colorimetric detection [addition of Pi reagent, *i.e.*, H_2_SO_4_ (2.5 M), ammonium molybdate (1%), antimony potassium tartrate (0.014%), acetic acid (20%), and freshly prepared ascorbic acid (1%), as well as subsequent measurement after 20 min using a Paradigm microplate reader (Beckman Coulter, Germany; 710 nm, room temperature)]. K_2_HPO_4_ was used for calibration purposes.

### Statistical analyses

Experiments were performed independently with at least two repeats. Effect values were put into relation of the reference inhibitor benzo[*d*][1,3]dioxol-5-ylmethyl)piperazin-1-yl)-6,7,8,9-tetrahydropyrimido-[4,5-*b*]indo-lizine-10-carbonitrile (ABCC1) [32], cyclosporine A (ABCB1), or Ko143 (ABCG2). Full-blown concentration-effect curves of the respective compounds and IC_50_ values were determined by non-linear regression using GraphPad Prism version 8.4.0 taking either three- or four-parameter logistic equations into account, whichever was statistically preferred. Significance was calculated using a t test (ABCC1 transport activity) or one-sample t test (ABCC1 ATPase activity; fictional value: 0) considering a *p* value of 0.05 as significant; *p* ≤ 0.05*; *p* ≤ 0.01**; *p* ≤ 0.001***; *p* < 0.001****.

## Supplementary Information

**Additional file 1.**

**Additional file 2.**

## Data Availability

Additional file 1 is freely available at (i) zenodo (10.5281/zenodo.13606805) [37]. (ii) github (including scripts; https://github.com/PANABC-INFO/CAPS_Rare-Modes-of-action). (iii) PANABC.info (http://www.panabc.info). Additional file 2 is available online on the journals’ homepage.

## Abbreviations

AC_50_: Half-maximal activation concentration
A_max_: Maximal activation
ATCC: American Type Culture Collection
ABC: ATP-biding cassette
CAS: Chemical abstract services
C@PA: Computer-aided pattern analysis
CLogP: Calculated octanol–water partition coefficient
CLogS: Calculated solubility
C@PS: Computer-aided pattern scoring
FBS: Fetal bovine sera
GSH: Reduced glutathione
H-bond: Hydrogen bond
MOE: Molecular operating environment
MOPS: [3-(N-morpholino)propanesulfonic acid
MR: Molar refractivity
MW: Molecular weight
SAReport: Structure–activity-report
TPSA: Topological polar surface area
