# Supplementary material for: Computer-aided pattern scoring (C@PS): a novel cheminformatic workflow to predict ligands with rare modes-of-action
Source: J Cheminform. 2024 Sep 23;16:108. doi: 10.1186/s13321-024-00901-5 (PMC11421111; doi:10.1186/s13321-024-00901-5)
Supplement: Supplementary file 2 — Additional file 2. [file 13321_2024_901_MOESM2_ESM.pdf]

Journal of Cheminformatics

METHODOLOGY **Open Access**

Computer-aided Pattern Scoring (C@PS): A Novel Cheminformatic Workflow to Predict Ligands with Rare Modes-of-action

Sven Marcel Stefan [1,2,3,†], Katja Stefan [1,2,†], and Vigneshwaran Namasivayam [2,4,*]

Affiliations

1. University of Oslo and Oslo University Hospital, Department of Pathology, Rikshospitalet, Sognsvannsveien 20, 0372 Oslo, Norway
2. University of Lübeck and University Medical Center Schleswig-Holstein, Lübeck Institute of Experimental Dermatology, Medical Systems Biology Division, Medicinal Chemistry and Systems Polypharmacology, Ratzeburger Allee 160, 23538 Lübeck, Germany
3. Medical University of Lublin, Department of Biopharmacy, Chodzki 4a, 20-093 Lublin, Poland
4. University of Bonn, Pharmaceutical Institute, Department of Pharmaceutical and Cellbiological Chemistry, An der Immenburg 4, 53121 Bonn, Germany

* corresponding author: Vigneshwaran Namasivayam (vnamasiv@uni-bonn.de)

† S.M.S & K.S. contributed equally to this work

Supplementary Information

Supplementary Figure S1……………………………………………………………………………………… P2

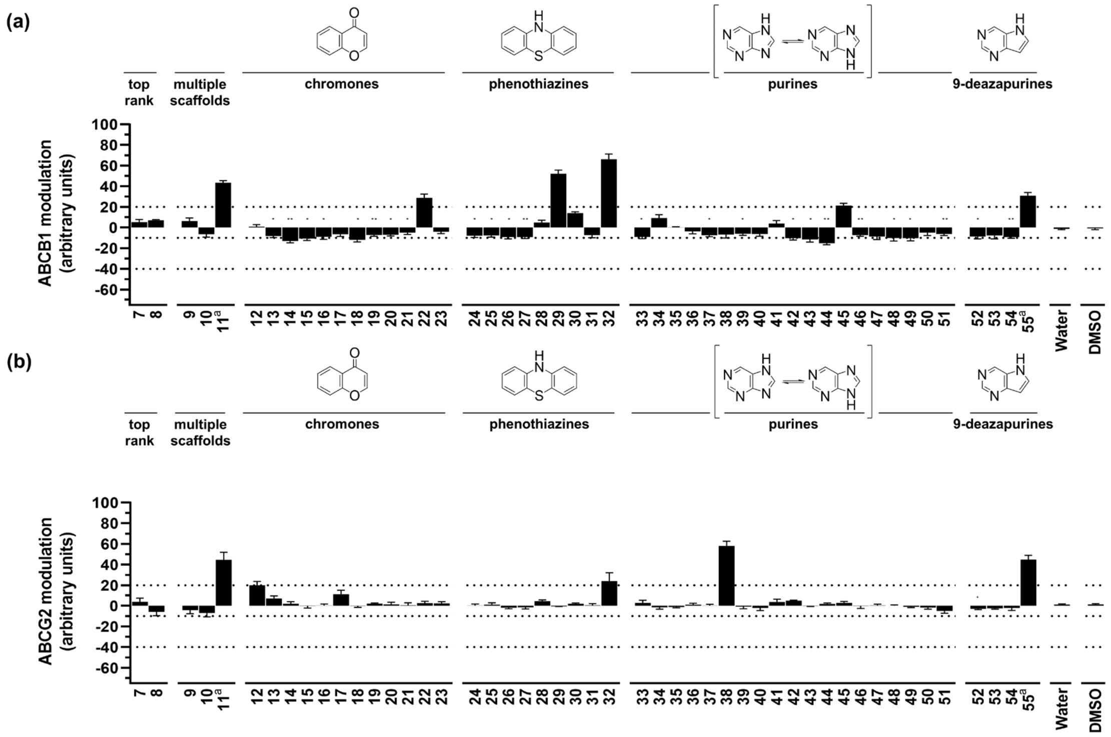

Supplementary Fig. S1 Screening of compounds **7**–**55** (10 µM) against ABCB1 (a) and ABCG2 (b) in daunorubicin (a) and pheophorbide A (b) assays using ABCB1-expressing A2780/ADR (a) and ABCG2-expressing MDCK II BCRP (b) cells, respectively, as described earlier [1]. Normalization of data was performed using the biological effects of the ABCB1 and ABCG2 reference inhibitors cyclosporine A and Ko143 (100%), respectively, and pure cell culture medium (0%). Shown are mean values ± standard error of the mean (SEM) of at least three independent experiments, and significance was calculated using a t test considering a p value of 0.05 as significant; $p \leq 0.05$; *; $p \leq 0.01$: **; $p \leq 0.001$: ***: $p < 0.001$: ****; [a] inhibitory activity of these compounds already reported previously [2]

1. Puri S, Stefan K, Khan SL, Pahnke J, Stefan SM, Juvale K (2023) Indole derivatives as new structural class of potent and antiproliferative inhibitors of monocarboxylate transporter 1 (MCT1; SLC16A1). J Med Chem 66:657. https://doi.org/10.1021/acs.jmedchem.2c01612
2. Namasivayam V, Stefan K, Gorecki L, Korabecny J, Soukup O, Jansson PJ, Pahnke J, Stefan SM (2022), Physicochemistry shapes bioactivity landscape of pan-ABC transporter modulators: Anchor point for innovative Alzheimer's disease therapeutics. Int J Biol Macromol 217:775. https://doi.org/10.1016/j.ijbiomac.2022.07.062
